# Advancing Influenza Prevention: The Case for Pre-Exposure Prophylaxis (PrEP)

**DOI:** 10.3390/vaccines14080666

**Published:** 2026-07-30

**Authors:** Hanumantha Rao Paritala, Luis Mier-y-Teran-Romero, Ramya Natarajan, Peter Adams, Cassandra Spector, Katherine Topf, Ashwin Kadambi, Julia A. Falvey, Mark J. Lamias, David P. Durham, Kimberly Armstrong

**Affiliations:** 1 Biomedical Advanced Research and Development Authority (BARDA), Administration for Strategic Preparedness and Response (ASPR), U.S. Department of Health and Human Services (HHS), Washington, DC 20024, USA; 2 Leidos, Supporting the Biomedical Advanced Research and Development Authority (BARDA), Administration for Strategic Preparedness and Response (ASPR), U.S. Department of Health and Human Services (HHS), Washington, DC 20024, USA; 3 Oak Ridge Institute for Science and Education, Supporting the Biomedical Advanced Research and Development Authority (BARDA), Administration for Strategic Preparedness and Response (ASPR), U.S. Department of Health and Human Services (HHS), Washington, DC 20024, USA; 4 Booz Allen Hamilton, Supporting the Biomedical Advanced Research and Development Authority (BARDA), Administration for Strategic Preparedness and Response (ASPR), U.S. Department of Health and Human Services (HHS), Washington, DC 20024, USA

**Keywords:** influenza pre-exposure prophylaxis, influenza PrEP, PrEP, chemoprophylaxis, long-acting antivirals, antivirals, pandemic preparedness, medical countermeasures, target product profile, agent-based modeling

## Abstract

Influenza remains a significant national health security threat, particularly for vulnerable populations, as existing control measures do not fully mitigate its impact. This gap in protection is especially pronounced early in a pandemic when a well-matched vaccine may not yet be available, as well as in populations unable to mount an optimal vaccine response. Clinical studies support pre-exposure prophylaxis (PrEP) therapeutics as a promising complementary strategy to reduce influenza transmission and disease severity, potentially easing the strain on healthcare systems during outbreaks. This manuscript outlines the Biomedical Advanced Research and Development Authority’s (BARDA’s) target product profile (TPP) for a long-acting influenza PrEP product, reviews the current development landscape, and models the potential impact of early PrEP product deployment using agent-based modeling in a synthetic population of 19.5 million people across pandemic scenarios resembling the 1918, 1968, and 2009 influenza pandemics. Simulations showed that early deployment of a 70%–effective PrEP reduced cumulative and peak infections, delayed the epidemic peak, and provided the greatest benefit in less transmissible pandemics; at 40–50% coverage, PrEP fully mitigated a 2009-like pandemic and substantially reduced transmission in 1918- and 1968-like scenarios. The TPP defines key characteristics of an effective PrEP option for seasonal and pandemic influenza that (1) demonstrates a strong safety and tolerability profile across all populations; (2) targets a direct-acting antiviral mechanism of action; (3) reduces the relative risk of symptomatic influenza infection by at least 70% in an unvaccinated population and (4) provides single-dose, season-long protection to optimize patient adherence. In this context, integrating PrEP into influenza prevention strategies could improve control of virus spread, strengthen protection for high-risk groups, and significantly reduce the overall public health impact of seasonal and pandemic influenza.

## 1. Introduction

Influenza is a highly contagious, acute viral respiratory infection primarily spread through respiratory droplets or direct contact. Its symptoms—such as fever, headache, muscle aches, sore throat, and cough—are clinically indistinguishable from those of other respiratory viral illnesses without laboratory confirmation. In some cases, and particularly among individuals at high risk, influenza can lead to severe complications or even death [1].

Influenza affects individuals across all age groups but poses the greatest risk of severe outcomes for vulnerable populations, including older adults, young children, and those with compromised immune systems. According to the Centers for Disease Control and Prevention (CDC), between the 2015–2016 and 2019–2020 influenza seasons, the United States experienced an estimated 24 to 41 million infections annually, resulting in up to 710,000 hospitalizations and 52,000 deaths per year [2]. Given its substantial impact on morbidity, mortality, and healthcare costs, influenza remains a significant public health challenge.

While seasonal influenza recurs each year, influenza pandemics are rare. A pandemic occurs when a novel influenza virus emerges that can spread efficiently among humans and where the global population has little to no pre-existing immunity [3]. Over the past century, there have been four major influenza pandemics—in 1918, 1957, 1968, and 2009—with the 1918 pandemic being the most severe in terms of global impact [4,5]. Influenza viruses undergo constant genetic changes through antigenic drift, which limits the duration of immunity acquired through infection or vaccination [4,6]. Annual immunization with vaccines that closely match the currently circulating viruses is the cornerstone of effective seasonal influenza prevention [7,8], but provides incomplete protection when viruses undergo antigenic drift after vaccines are formulated. In contrast, traditional pandemic vaccine production takes 6 to 8 months from candidate strain recommendation, or pandemic recognition, to actual production and delivery, creating a gap in protection [9,10]. Given that current strategies for vaccination against seasonal and pandemic influenza vaccines provide incomplete protection, new or improved strategies are needed to safeguard public health.

This study describes the Biomedical Advanced Research and Development Authority’s (BARDA’s) target product profile (TPP) for a long-acting influenza pre-exposure prophylaxis (PrEP) product, reviews the current development landscape, and uses agent-based modelling to estimate the potential population-level impact of early PrEP deployment during influenza pandemics with differing levels of transmissibility.

## 2. Rationale for Influenza PrEP

Pre-exposure prophylaxis (PrEP) intended for general use in the US population offers a promising strategy for bridging the gap between the declaration of a public health emergency and the availability of a well-matched vaccine. PrEP involves using drugs to limit infection and the spread of disease during periods of heightened viral activity. In the context of HIV prevention, PrEP has historically proven to be an effective strategy for preventing infection among individuals who have a substantial risk of exposure. In addition, experience with HIV PrEP dosing regimens has informed the development of improved therapeutics with reduced dosing frequency, and clinical studies have shown that high levels of PrEP adherence reduce the risk of HIV infection by 70% [11].

During the COVID-19 pandemic, Evusheld^TM^ [12] and Pemgarda^®^ [13] received emergency use authorization (EUA) as COVID-19 PrEP therapeutics. Evusheld^TM^ was authorized for use as PrEP in adults and children over age 12 who were immunocompromised and may not have been able to mount an adequate response to COVID-19 vaccines and for those in whom vaccination was not recommended [14]. A meta-analysis of the clinical trial data from studies addressing clinical effectiveness of Evusheld^TM^ found that it reduced breakthrough infection by 40.5% and mortality by 92.4% for immunocompromised patients [15]. However, Evusheld^TM^ was discontinued due to reduced effectiveness against circulating variants. Pemgarda^®^ remains authorized for use as an intravenous monoclonal antibody for immunocompromised individuals aged 12+ administered approximately every three months [13]. The combined experience with HIV and COVID-19 underscores the effectiveness of PrEP therapeutic-based interventions and supports their inclusion as an integral part of the pandemic preparedness strategy for influenza.

Given the constraints on manufacturing (e.g., time-intensive processes) and challenges with stockpiling (e.g., shelf-life duration and storage requirements), medical countermeasure distribution during a pandemic must be prioritized to protect highly exposed and vulnerable populations, such as first responders [16], healthcare workers [17], and residents of long-term care facilities [18]. In the absence of an effective vaccine, first responders and healthcare workers are at increased risk of infection, which can exacerbate worker shortages and strain healthcare system capacity [19]. Residents of long-term care facilities are particularly susceptible to rapid transmission and severe outcomes given their advanced age, comorbidities, and congregate living settings. This was evident during the COVID-19 pandemic when long-term care facility residents accounted for 10% of all cases and 44% of all deaths [20]. Thus, early prioritization of these populations for protective measures is essential to minimize severe outcomes, limit transmission, and sustain workforce operations during outbreaks.

In addition to the general use by the US population, a PrEP option would also provide significant benefits as an adjunct to vaccines for seasonal influenza. In the United States, an estimated 6.2% of adults aged 18–64 years have immunosuppressive conditions [21,22], and 41.6% of adults aged 65 years and older are affected by at least one immunocompromising condition [23]. In these groups, influenza continues to cause substantial morbidity and mortality, underscoring the need for alternative preventive strategies, such as PrEP [24]. Therefore, it would be beneficial to have a PrEP option for populations that cannot achieve sufficient immunity after seasonal influenza vaccination.

Currently, there are four therapeutics approved by the U.S. Food and Drug Administration (FDA) to treat influenza, two of which (oseltamivir phosphate and zanamivir) are also FDA-approved for use as PrEP for influenza. Clinical trials evaluating seasonal PrEP studies have demonstrated that oseltamivir and zanamivir significantly reduce the odds of laboratory-confirmed symptomatic influenza by 74% and 69%, respectively [25]. When utilized for PrEP during community outbreaks, oral oseltamivir phosphate is approved for up to 6 weeks during a community outbreak [26], whereas inhaled zanamivir is indicated for daily dosing over a 28-day period [27,28]. Daily dosing regimens pose significant obstacles that hinder the consideration of either antiviral as a practical option for use in pandemic preparedness, including challenges with patient compliance, maintaining adequate stockpiles, and coordinating the sustained distribution of these therapeutics.

Prior experience with PrEP for HIV has shown that while PrEP can be highly effective, compliance is a significant factor impacting the success of PrEP therapeutics. For example, Truvada^®^ requires consistent, repeated dosing over a defined period [11], and suboptimal patient adherence reduced its effectiveness as an HIV prophylactic [11]. Apretude, a long-acting injectable dosed intramuscularly every 2 months, has demonstrated efficacy for HIV PrEP [29]. More recently, Yeztugo^®^ was approved with twice-yearly dosing and showed superiority to the daily dosing regimen with Truvada^®^ [30]. These studies highlight the importance of developing PrEP strategies with reduced dosing regimens to enhance patient compliance.

## 3. Target Product Profile (TPP) for Influenza PrEP Therapeutic

BARDA’s overall objective is to partner in the development of PrEP therapeutics for both pandemic and seasonal influenza, with the goal of achieving FDA approval. Safety is an important consideration both in terms of maximizing accessibility for all demographics and minimizing the risk of adverse outcomes in an otherwise healthy population. Drawing on precedents of PrEP therapeutics for other indications, BARDA has established a target product profile (TPP) for an influenza PrEP therapeutic (Table 1) that addresses key challenges of patient compliance with daily dosing regimens and accounts for anticipated demands on manufacturing capacity following the declaration of a pandemic.

In addition to the established experience with HIV PrEP products, we evaluated the population-wide application of PrEP in an influenza pandemic response, supporting its potential role as a scalable preventive intervention in multiple pandemic scenarios. Viral transmission modeling using an agent-based framework was employed to evaluate the potential population-level impact of the TPP-proposed PrEP product on cumulative influenza infections during a pandemic. Using census tract-level resolution, we divided a synthetic population for the greater New York City area (around 19.5 million total population as of 2017) into three age categories (<18, 18–64 and 65+ years) to reflect real-world implementation of PrEP early in a pandemic prior to vaccine availability. We evaluated three scenarios of viral transmissibility using literature estimates for basic reproductive numbers from the 1918, 1968, and 2009 influenza pandemics in order of decreasing transmission intensity. We also considered scenarios with varying target product inventory sizes covering 0–50% of the population (in increments of 10%). PrEP is distributed randomly across the population without consideration of age or any other risk factor. We do not account for variables influencing uptake such as the occurrence of adverse events or distribution logistics. Consistent with the optimal profile described in Table 1, the desired target PrEP product is assumed to have an efficacy of 70% against symptomatic influenza illness in a vaccinated population. Differential efficacy across the population and the emergence of viral strains resistant to treatment are not considered. For context and benchmarking purposes only, we reference a hypothetical vaccine efficacy of 58% against symptomatic influenza (derived from 40% efficacy against infection and 30% against symptoms given infection) [31,32]. The vaccine is not modeled as a concurrently deployed intervention in the primary analysis but serves as an anchor to situate PrEP performance relative to an established standard. We assume that the product is distributed to the population early in the pandemic, when a negligible fraction of the population is infected, and the product efficacy does not wane over a single epidemic wave or season. A more detailed description of the model, the selected parameter values, and additional simulations combining target product and vaccine distribution can be found in the Supplementary Materials.

**Figure 1:**
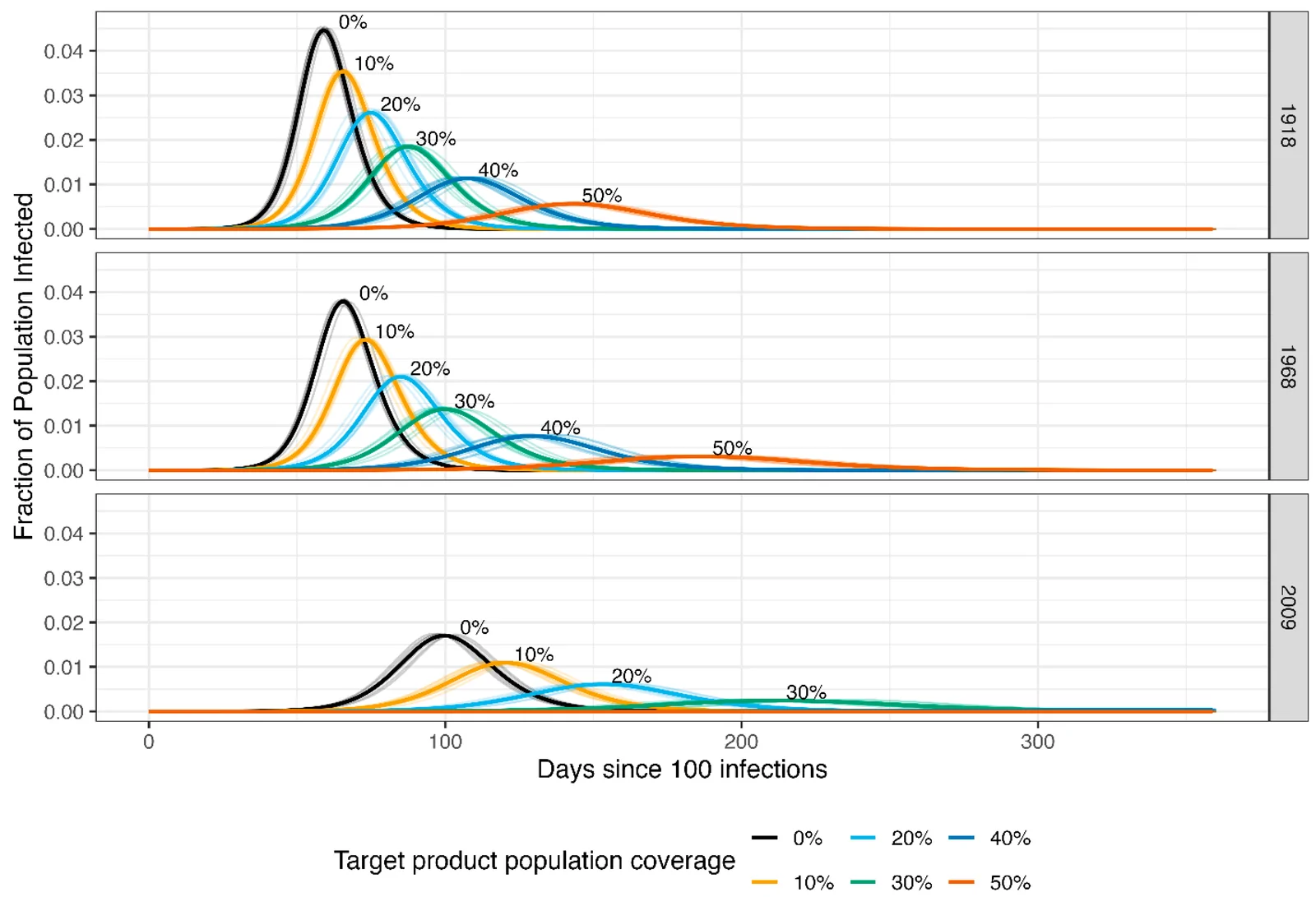
Simulated influenza epidemic curves showing the impact of mitigation from early PrEP product deployment. Each panel corresponds to a different value of the basic reproductive number—chosen to match estimates for the 1918, 1968, and 2009 influenza pandemics—while the colored lines correspond to varying sizes of PrEP product inventory covering the indicated percentages of the population. Each epidemic curve is a stochastic realization for a combination of basic reproductive number and inventory size; the average of ten stochastic realizations is shown as a thick curve. Note that for the bottom panel, product inventories of 40% and 50% lead to nearly complete mitigation of viral transmission.

Figure 1 illustrates epidemic waves as a function of time for varying scenarios of viral transmissibility and target product inventory size. Early deployment of PrEP therapeutics yields three primary public health benefits relative to the unmitigated or unvaccinated baseline: (i) a decreased cumulative number of infections (proportional to the area under each epidemic curve); (ii) a decreased number of infections at peak; and (iii) a delayed epidemic peak. The impact of the medical countermeasure is greater for less transmissible viral strains. For example, in the case of a virus with a level of transmissibility matching that of the 2009 pandemic, product inventory sizes covering 40% and 50% of the population are sufficient for total mitigation. In contrast, under 1918- or 1968-like transmissibility, the same coverage levels substantially suppress but do not eliminate epidemic waves.

**Figure 2:**
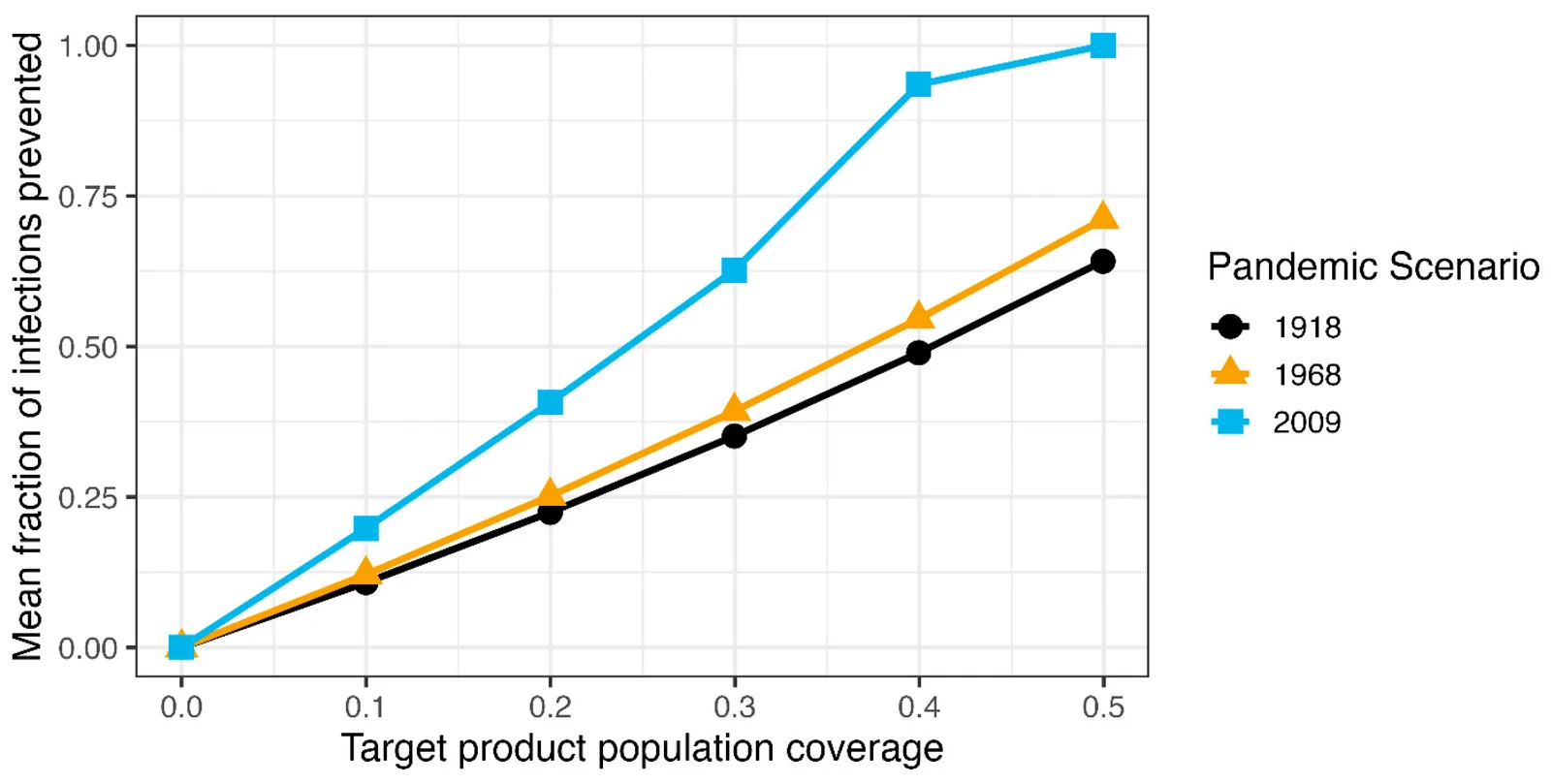
Mean fraction of infections averted relative to a baseline without any PrEP product deployment, as a function of the fraction of the population receiving the product.

To summarize the overall impact of PrEP product distribution more succinctly, Figure 2 presents the mean fraction of total infections prevented through PrEP therapeutic deployment relative to an unvaccinated baseline. This figure demonstrates that broader population coverage leads to progressively greater reductions in infections, with stronger proportional effects observed in lower-transmissibility scenarios.

Although not directly addressed by the modeling here, evidence from HIV PrEP programs and influenza modeling studies suggests PrEP for influenza could reduce transmission at the individual level, particularly in high-risk groups, and lower susceptibility at the population level to mitigate seasonal outbreaks and pandemic impact. Oseltamivir phosphate and zanamivir have shown efficacy for PrEP, but their required daily dosing limits practicality. In contrast, long-acting antivirals and monoclonal antibodies offer more feasible alternatives [33]. Given delays in well-matched vaccine availability in a pandemic, rapidly deployable PrEP may protect frontline workers and vulnerable populations while reducing healthcare system strain and overall disease burden.

## 4. Landscape Analysis of Potential PrEP Therapeutics

PrEP for influenza remains an underutilized yet critically important strategy in the prevention of seasonal and pandemic influenza outbreaks, particularly for high-risk and highly exposed populations such as the elderly, immunocompromised individuals, and healthcare workers. Antigenic drift, suboptimal vaccine uptake, and varying vaccine effectiveness each season have led to sustained interest in chemoprophylaxis as a supplemental approach.

### 4.1. Small Molecule Antivirals

As the most widely studied antiviral used for prophylaxis, the neuraminidase inhibitor (NAI) oseltamivir phosphate has shown substantial efficacy in community outbreak prophylaxis when administered as a daily oral regimen for up to 6 weeks [26]. In a pivotal trial, Welliver et al. reported an 89% reduction in laboratory-confirmed influenza among individual contacts and an 84% reduction among household contacts [34]. However, the utility of oseltamivir phosphate for pandemic PrEP is limited due to its frequent dosing requirement, highlighting the need for novel prophylactic agents with extended duration of protection and simplified dosing regimens to improve compliance and expand applicability in both seasonal and pandemic contexts.

Zanamivir, another NAI, is delivered via inhalation and shares a similar mechanism of action and efficacy profile with oseltamivir phosphate. However, its use is limited by delivery constraints, particularly in individuals with underlying pulmonary conditions. The inhaled route, while effective, poses logistical challenges, particularly in pediatric or elderly populations who may struggle with inhaler use or adherence to daily dosing schedules.

Currently, there is insufficient clinical evidence to support the use of peramivir [35], baloxavir marboxil [36], or laninamivir [36] for influenza PrEP. Peramivir is impractical in a pandemic setting due to its intravenous route of administration and the burden to the healthcare system of dosing this antiviral. Laninamivir, though effective as post-exposure prophylaxis, lacks data for pre-exposure use. Baloxavir marboxil has demonstrated potential in reducing secondary transmission of influenza [37]; however, baloxavir marboxil is not approved for PrEP as there is no available safety and efficacy data in pregnancy and in severely immunocompromised patients [38], and its use may be limited by clinically significant drug-cation interactions. Baloxavir forms chelates with polyvalent cations (e.g., calcium, aluminum, magnesium, iron, selenium and zinc), and co-administration with products containing these ions results in a significant decrease in baloxavir exposure in nonhuman primates [38,39].

### 4.2. Monoclonal Antibodies

Despite efforts to develop anti-influenza mAbs, none have been authorized or approved for influenza prophylaxis or treatment, likely due to their insufficient distribution within the primary site of infection in the upper respiratory tract, limited clinical efficacy, and/or challenges in demonstrating meaningful benefit over oral antiviral therapies. For example, the broadly neutralizing anti-hemagglutinin (HA) mAb MEDI8852 was safe but did not improve clinical or virologic outcomes over standard therapy in uncomplicated influenza, limiting its clinical value for this population [40]. Likewise, other anti-HA mAbs, including CR6261 and CR8020, showed promise when tested in combination in in vivo studies but failed to show clinical benefit in humans [41]. Notably, VIR-2482 is the only broad-spectrum anti-HA mAb targeting seasonal and pandemic influenza A to enter phase 2 clinical trials for PrEP. VIR-2482 is an engineered derivative of MEDI8852 that was specifically designed with an “LS mutation” (M428L/N434S) in the Fc domain of the heavy chain to extend its half-life for prophylactic use. Though VIR-2482 was well-tolerated in the phase 2 trial, it did not meet its primary endpoint in reducing the incidence of influenza-like illness in healthy, unvaccinated adult subjects at either 450 mg or 1200 mg doses administered intramuscularly [33,42].

Recently, a study investigating intranasal administration of CR9114, another mAb targeting the stem domain of HA, has demonstrated protective efficacy from lethal doses of influenza A (H5N1) in two separate murine studies [43,44]. The mechanism of action for CR9114 in these studies was found to be mediated in an Fc-independent manner, which was unique to the intranasal route of administration.

### 4.3. Drug-Fc Conjugate for Influenza PrEP

CD388 is a multivalent conjugate of dimerized zanamivir with the N-terminal half-life-extended Fc domain of human IgG1. While zanamivir alone has not demonstrated superiority to oseltamivir phosphate in preclinical studies, CD388 exhibited broad activity and long-acting protection (~3 weeks) that was not achieved by oseltamivir against pandemic and seasonal influenza strains [45]. Two phase 1 trials to assess safety and dose escalation tolerability of CD388 have had positive results [46]. Also, in a randomized controlled phase 2a human challenge study, CD388 demonstrated prophylactic activity against influenza infection [47]. Additionally, the phase 2b NAVIGATE trial plans to evaluate the protective efficacy of CD388 when administered as a single subcutaneous injection in healthy adults not at risk for influenza complications (NCT06609460).

While no small molecule antiviral agent or mAb currently holds full regulatory approval in the U.S. explicitly for long term (≥6 months) PrEP against influenza (Table 2), regulatory precedence has been established through the approval of long-acting antivirals for HIV PrEP.

## 5. Discussions and Conclusions

Influenza remains a major global health challenge, causing significant morbidity and mortality annually. Despite advances in biomedical science, the fluctuating efficacy of vaccines and the underutilization of antiviral medications continue to complicate prevention and treatment efforts. Influenza virus’s rapid mutation rates necessitate constant vigilance and adaptation of prevention strategies.

While vaccination stands as the frontline measure for influenza prevention, the process of annual vaccine strain selection is scientifically and logistically challenging. Ensuring the vaccine strains match the circulating strains and distributing the resulting vaccine on time is critical for countering the rapid mutation of influenza viruses [49,50]. However, vaccination policies and coverage vary globally [51,52]. In addressing the challenges associated with influenza prevention, a notable gap exists between the effectiveness of current vaccines and the absence of long-acting PrEP options. This disconnect limits sustained protection, particularly among high-risk populations. Consequently, this gap significantly undermines the overall effectiveness of influenza prevention strategies.

One promising solution to bridge this divide is the utilization of prophylactic antivirals [37,45]. Antivirals targeting the conserved elements of the influenza virus may offer a robust alternative to traditional vaccines, particularly when vaccine effectiveness is compromised by mismatched circulating strains or rapid antigenic shifts. The potential of prophylactic antivirals to aid influenza prevention efforts increases substantially when they can provide protection throughout the entire influenza season with minimal dosing requirements. Such a strategy not only addresses vaccine strain variability that reduces vaccine effectiveness but also provides an alternate solution for the gap in protection, particularly for vulnerable and highly exposed populations, thereby improving public health outcomes.

The TPP in Table 1 outlines an influenza PrEP product that could be used for mitigation of seasonal influenza disease and influenza pandemic preparedness. The product must exhibit a favorable safety and tolerability profile across all demographic groups, ensuring minimal side effects. Furthermore, as a minimal requirement, it should demonstrate at least 70% relative risk reduction in symptomatic influenza when compared to an unvaccinated population. Optimally, a 70% relative risk reduction in influenza symptoms relative to a vaccinated population is desired (Table 1). To improve patient compliance and reduce the burden of repeat dosing, the product should be designed to offer protection throughout the entire influenza season with a single dose. Achieving these targets will ensure the product can address significant gaps in current seasonal influenza prevention strategies and support public health preparedness.

At present, antiviral medications play a critical role in the management of influenza, serving as both prophylactic and therapeutic interventions. The Infectious Disease Society of America (IDSA) has published comprehensive guidelines outlining the use of antiviral agents to effectively prevent or treat influenza infections [53]. These guidelines recommend timely administration of antivirals, particularly for populations that do not achieve protection after seasonal influenza vaccination. The prophylactic use of antivirals is particularly recommended in settings where vaccine effectiveness is low or when vaccination is not possible. Additionally, therapeutic use of antivirals is encouraged for confirmed cases of influenza to reduce the duration of symptoms and prevent serious complications [50,54].

Looking ahead, the integration of PrEP therapeutics into influenza prevention strategies has the potential to meaningfully reshape both seasonal and pandemic influenza control. Importantly, influenza PrEP should be considered a complementary strategy rather than a substitute for vaccination. Robust influenza vaccination programs must remain the cornerstone of influenza prevention, particularly for vulnerable populations at increased risk of severe disease. Within this framework, pharmacological prophylaxis has the potential to provide additional protection for individuals at high risk of exposure or those who may not mount an adequate immune response to vaccination, thereby complementing existing vaccination efforts.

For seasonal influenza, PrEP could substantially reduce disease burden among populations at highest risk of severe outcomes—particularly older adults and immunocompromised individuals who have suboptimal protection from vaccination alone. In a pandemic, when vaccine availability is delayed and healthcare systems are strained, deployment of long-acting PrEP during the early phases of an outbreak could blunt transmission, reduce peak incidence, and protect essential workers and vulnerable populations. Together with vaccination, scalable and durable influenza PrEP strategies offer a forward-leaning approach to preparedness, strengthening public health resilience against both predictable seasonal epidemics and unpredictable pandemic threats.

## Figures and Tables

**Table 1:** BARDA’s Target Product Profile for PrEP Therapeutic.

Product Properties	Minimal Attributes	Optimal Attributes
Mechanism of Action	Direct-acting antiviral (targets highly conserved viral factors)	Direct-acting antiviral (targets highly conserved viral factors)
Clinical Efficacy	70% relative risk reduction in symptomatic influenza infection when comparing treated vs. placebo-treated patients in an unvaccinated population	70% relative risk reduction in symptomatic influenza infection when comparing treated vs. placebo-treated patients in a vaccinated population
Target Population	General use in people who achieve insufficient protection after seasonal influenza vaccination For pandemic response, demonstrated benefit in otherwise healthy populations in addition to those above	General use in people who achieve insufficient protection after seasonal influenza vaccination For pandemic response, demonstrated benefit in otherwise healthy populations in addition to those above
Therapeutic Modality	Monoclonal antibodies (mAbs), other biologics, long-acting small molecules, among others	mAbs, other biologics, long-acting small molecules, among others
Dose Regimen and Route of Administration	Single dose by any route of administration including intravenous	Single dose by oral, transdermal, or injectable (intramuscular or subcutaneous) using volumes suitable for a single injection
Onset of Protection	7 days or less	Immediately following administration
Duration of Protection	One month from a single dose	Six months from a single dose
Safety and Tolerability	Safe and well tolerated for general use prevention indication	Safe and well tolerated for general use prevention indication
Drug Interactions/Contraindications	Some drug–drug interactions (DDIs) and contraindications tolerated; manageable risk	No DDIs or contraindications
Clinical Pharmacology	C_min_ > EC_50_ (protein-binding adjusted) for the entire dosing interval OR rationale for alternate pharmacokinetic/pharmacodynamic profile	C_min_ > EC_90_ (protein-binding adjusted) for the entire dosing interval
Stability and Storage	Storage and shipping at −20 °C, 2–8 °C, or room temperature; stability ≥ 2 years	Storage and shipping at room temperature; stability ≥ 5 years
Manufacturing and Scalability	Manufacturing process is scalable for 5 million doses per year	Manufacturing process is scalable for 5 million doses per month; product is manufactured in the U.S.
Manufacturing Cost of Goods	Commercially viable in the seasonal influenza vaccine market	Competitive in the seasonal influenza vaccine market

**Table 2:** Influenza Antivirals for PrEP: Dosing and Approval Status.

Drug Class	Drug	PrEP Dose and Route of Administration	PrEP Approval Status/Comments
Neuraminidase inhibitors	Oseltamivir phosphate [26]	Ages 3 months to <1 year: 3 mg/kg, once daily for 7 days, oral route. Ages 1–12 years: 30–75 mg (based on weight), once daily for 10 days, oral route. In community outbreak: (based on weight) for up to 6 weeks. Ages ≥ 13 years: 75 mg, once daily for 10 days, oral route. In community outbreak: 75 mg, once daily for up to 6 weeks.	FDA-approved for chemoprophylaxis in patients ages ≥ 1 year. Dosage adjustment is needed based on the patient’s weight and creatinine clearance rate.
	Zanamivir [28]	Household setting: 10 mg, once daily for 10 days. Community outbreaks: 10 mg, once daily for 28 days. The 10 mg dose is provided by two inhalations.	FDA-approved for chemoprophylaxis. Contraindicated in patients with lactose/milk protein allergy or underlying airway disease
	Peramivir	—	Not FDA-approved for PrEP
	Laninamivir	—	Not FDA-approved for PrEP
Ion channel inhibitors	Amantadine [48]	Ages 1–9 years: 5 mg/kg, daily in two divided doses (not to exceed 150 mg/day), oral route. Ages ≥ 10 years: 100 mg, twice daily (not to exceed 200 mg/day) Children < 40 kg: dosing can be 5 mg/kg, daily, oral route.	FDA-approved for PrEP but no longer recommended due to widespread resistance
	Rimantadine [48]	Ages 1–9 years: 5 mg/kg, daily in one or two divided doses (not to exceed ~150 mg/day), oral route. Ages ≥ 10 years: 100 mg, twice daily (not to exceed 200 mg/day), oral route.	FDA-approved for PrEP but no longer recommended due to resistance
Cap-dependent endonuclease inhibitor	Baloxavir marboxil [39]	—	Not FDA-approved for PrEP
Polymerase inhibitor	Favipiravir	—	Not FDA-approved for PrEP
Monoclonal antibodies	MEDI8852, VIR-2486, CR6261, CR8020, CR-9114	—	Under development for PrEP
Drug-Fc conjugate	CD388	Once per season subcutaneous 450 mg dose is being studied in phase 3 (NCT07159763) in those 12 years of age and older	Under development for PrEP

An em dash (—) indicates that no dosage information was available for PrEP.

## Data Availability

Data is contained within the article or Supplementary Materials—The original contributions presented in this study are included in the article/Supplementary Materials. Further inquiries can be directed to the corresponding author.

## Supplementary Materials

The following supporting information can be downloaded at https://www.mdpi.com/article/10.3390/vaccines14080666/s1: Agent-based modeling, Supplementary Table S1: Parameter assumptions, Supplementary Table S2: infectious contact rate by age group and pandemic scenario, Supplementary Table S3: Medical countermeasure (MCM) parameter assumptions, and Figure S1: fraction of infections averted, relative to an unmitigated baseline, as a function of TPP coverage (x-axis), vaccine coverage (panels along columns), virus transmissibility scenario (panels along rows) and by how they are attributed by MCM (colors and marker shape).

## References

[1] U.S. Centers for Disease Control and Prevention Influenza (Flu). https://www.cdc.gov/flu/signs-symptoms/index.html.

[2] U.S. Centers for Disease Control and Prevention Flu Burden: About Estimated Flu Burden. https://www.cdc.gov/flu-burden/php/about/index.html#:~:text=While%20the%20burden%20of%20flu,the%20United%20States%20each%20year.&text=CDC%20estimates%20that%20flu%20has,annually%20between%202010%20and%202024.

[3] U.S. Centers for Disease Control and Prevention Pandemic Flu: About Pandemic Influenza. https://www.cdc.gov/pandemic-flu/basics/index.html.

[4] Morse S.S. (1997). The Public Health Threat of Emerging Viral Disease. J. Nutr..

[5] Kilbourne E.D. (2006). Influenza Pandemics of the 20th Century. Emerg. Infect. Dis..

[6] U.S. Centers for Disease Control and Prevention Influenza (Flu): How Flu Viruses Can Change: “Drift” and “Shift”. https://www.cdc.gov/flu/php/viruses/change.html#:~:text=When%20a%20flu%20virus%20has,and%20fighting%20against%20the%20virus.

[7] Paules C.I., Sullivan S.G., Subbarao K., Fauci A.S. (2018). Chasing Seasonal Influenza—The Need for a Universal Influenza Vaccine. N. Engl. J. Med..

[8] U.S. Centers for Disease Control and Prevention Influenza (Flu): Key Facts About Seasonal Flu Vaccine. https://www.cdc.gov/flu/vaccines/keyfacts.html.

[9] Tosh P.K., Jacobson R.M., Poland G.A. (2010). Influenza Vaccines: From Surveillance Through Production to Protection. Mayo Clin. Proc..

[10] Rockman S., Laurie K. (2025). Understanding the Implications of Delaying Seasonal Influenza Vaccine Recommendations: An Industry Perspective. Vaccines.

[11] Fonner V.A., Dalglish S.L., Kennedy C.E., Baggaley R., O’reilly K.R., Koechlin F.M., Rodolph M., Hodges-Mameletzis I., Grant R.M. (2016). Effectiveness and safety of oral HIV preexposure prophylaxis for all populations. AIDS.

[12] Levin M.J., Ustianowski A., De Wit S., Launay O., Avila M., Templeton A., Yuan Y., Seegobin S., Ellery A., Levinson D.J. (2022). Intramuscular AZD7442 (Tixagevimab–Cilgavimab) for Prevention of COVID-19. N. Engl. J. Med..

[13] Wolfe C.R., Cohen J., Mahoney K., Holmes A., Betancourt N., Gupta D., Tosh K., Narayan K., Campanaro E., Katz C. (2025). Safety and Efficacy of Pemivibart, a Long-Acting Monoclonal Antibody, for Prevention of Symptomatic COVID-19: Interim Results from a Phase 3 Randomized Clinical Trial (CANOPY). Clin. Infect. Dis..

[14] Kmietowicz Z. (2021). COVID-19: Monoclonal antibodies authorised in US as alternative to vaccines for certain groups. BMJ.

[15] Suribhatla R., Starkey T., Ionescu M.C., Pagliuca A., Richter A., Lee L.Y.W. (2023). Systematic review and meta-analysis of the clinical effectiveness of tixagevimab/cilgavimab for prophylaxis of COVID-19 in immunocompromised patients. Br. J. Haematol..

[16] Nguyen L.H., Drew D.A., Graham M.S., Joshi A.D., Guo C.-G., Ma W., Mehta R.S., Warner E.T., Sikavi D.R., Lo C.-H. (2020). Risk of COVID-19 among front-line health-care workers and the general community: A prospective cohort study. Lancet Public Health.

[17] Fell A. (2020). SARS-CoV-2 Exposure and Infection Among Health Care Personnel—Minnesota, March 6–July 11, 2020. Morb. Mortal. Wkly. Rep..

[18] Yi S.H., See I., Kent A.G., Vlachos N., Whitworth J.C., Xu K., Gouin K.A., Zhang S., Slifka K.J., Sauer A.G. (2020). Characterization of COVID-19 in Assisted Living Facilities—39 States, October 2020. Morb. Mortal. Wkly. Rep..

[19] U.S. Fire Administration, FEMA Information for First Responders on Maintaining Operational Capabilities During a Pandemic. https://www.usfa.fema.gov/downloads/pdf/publications/first_responder_pandemic_operational_capabilities.pdf.

[20] Konetzka R.T., White E.M., Pralea A., Grabowski D.C., Mor V. (2021). A systematic review of long-term care facility characteristics associated with COVID-19 outcomes. J. Am. Geriatr. Soc..

[21] Patel M., Chen J., Kim S., Garg S., Flannery B., Haddadin Z., Rankin D., Halasa N., Talbot H.K., Reed C. (2020). Analysis of MarketScan Data for Immunosuppressive Conditions and Hospitalizations for Acute Respiratory Illness, United States. Emerg. Infect. Dis..

[22] U.S. Fire Administration, FEMA National First Responders Day: Honoring Our Everyday Heroes. https://www.usfa.fema.gov/blog/national-first-responders-day/.

[23] Grant L.R., Meche A., McGrath L., Miles A., Alfred T., Yan Q., Chilson E. (2023). Risk of Pneumococcal Disease in US Adults by Age and Risk Profile. Open Forum Infect. Dis..

[24] Wen S., Wu Z., Zhong S., Li M., Shu Y. (2021). Factors influencing the immunogenicity of influenza vaccines. Hum. Vaccines Immunother..

[25] Cooper N.J., Sutton A.J., Abrams K.R., Wailoo A., Turner D., Nicholson K.G. (2003). Effectiveness of neuraminidase inhibitors in treatment and prevention of influenza A and B: Systematic review and meta-analyses of randomised controlled trials. BMJ.

[26] FDA Highlights of Prescribing Information—TAMIFLU^®^ (Oseltamivir Phosphate) Capsules, for Oral Use; TAMIFLU^®^ (Oseltamivir Phosphate) for Oral Suspension. FDA. https://www.accessdata.fda.gov/drugsatfda_docs/label/2019/021087s071,021246s054lbl.pdf.

[27] Monto A.S., Robinson D.P., Herlocher M.L., Hinson J.J.M., Elliott M.J., Crisp A. (1999). Zanamivir in the Prevention of Influenza Among Healthy Adults. JAMA.

[28] FDA RELENZA—Highlights of Prescribing Information. FDA.

[29] FDA (2025). Highlights of Prescribing Information—APRETUDE. https://www.accessdata.fda.gov/drugsatfda_docs/label/2025/215499s009lbl.pdf.

[30] Kelley C.F., Acevedo-Quiñones M., Agwu A.L., Avihingsanon A., Benson P., Blumenthal J., Brinson C., Brites C., Cahn P., Cantos V.D. (2025). Twice-Yearly Lenacapavir for HIV Prevention in Men and Gender-Diverse Persons. N. Engl. J. Med..

[31] Basta N.E., Halloran M.E., Matrajt L., Longini I.M. (2008). Estimating Influenza Vaccine Efficacy from Challenge and Community-based Study Data. Am. J. Epidemiol..

[32] Belongia E.A., Simpson M.D., King J.P., Sundaram M.E., Kelley N.S., Osterholm M.T., McLean H.Q. (2016). Variable influenza vaccine effectiveness by subtype: A systematic review and meta-analysis of test-negative design studies. Lancet Infect. Dis..

[33] Plotnik D., Sager J.E., Aryal M., Fanget M.C., Peter A., Schmid M.A., Cebrik D., Mogalian E., Boundy K., Yeh W.W. (2024). A phase 1 study in healthy volunteers to investigate the safety, tolerability, and pharmacokinetics of VIR-2482: A monoclonal antibody for the prevention of severe influenza A illness. Antimicrob. Agents Chemother..

[34] Welliver R., Monto A.S., Carewicz O., Schatteman E., Hassman M., Hedrick J., Jackson H.C., Huson L., Ward P., Oxford J.S. (2001). Effectiveness of Oseltamivir in Preventing Influenza in Household Contacts: A Randomized Controlled Trial. JAMA.

[35] Barroso L., Treanor J., Gubareva L., Hayden F.G. (2005). Efficacy and Tolerability of the Oral Neuraminidase Inhibitor Peramivir in Experimental Human Influenza: Randomized, Controlled Trials for Prophylaxis and Treatment. Antivir. Ther..

[36] Ikematsu H., Hayden F.G., Kawaguchi K., Kinoshita M., de Jong M.D., Lee N., Takashima S., Noshi T., Tsuchiya K., Uehara T. (2020). Baloxavir Marboxil for Prophylaxis against Influenza in Household Contacts. N. Engl. J. Med..

[37] Fujii A., Oishi T., Kondo E., Morihara Z., Ikeda Y., Sumida H., Ninomiya Y., Uesugi S., Okazaki K., Yoshioka D. (2025). Efficacy of baloxavir marboxil as a prophylactic against influenza among in-patients and analysis of influenza virus mutations in their isolates at a Japanese hospital during the 2023–2024 flu season. J. Pharm. Health Care Sci..

[38] CDC (2026). Influenza Antiviral Medications: Summary for Clinicians. CDC. https://www.cdc.gov/flu/hcp/antivirals/summary-clinicians.html?CDC_AAref_Val=https://www.cdc.gov/flu/professionals/antivirals/summary-clinicians.htm.#:~:text=A%20randomized%20clinical%20trial%20reported,with%20an%20individual%20with%20influenza.

[39] FDA Highlights of Prescribing Information: XOFLUZA^®^ (Baloxavir Marboxil) Tablets, for Oral Use XOFLUZA^®^ (Baloxavir Marboxil) for Oral Suspension. https://www.gene.com/download/pdf/xofluza_prescribing.pdf.

[40] Ali S.O., Takas T., Nyborg A., Shoemaker K., Kallewaard N.L., Chiong R., Dubovsky F., Mallory R.M. (2018). Evaluation of MEDI8852, an Anti-Influenza A Monoclonal Antibody, in Treating Acute Uncomplicated Influenza. Antimicrob. Agents Chemother..

[41] Bangaru S., Zhang H., Gilchuk I.M., Voss T.G., Irving R.P., Gilchuk P., Matta P., Zhu X., Lang S., Nieusma T. (2018). A multifunctional human monoclonal neutralizing antibody that targets a unique conserved epitope on influenza HA. Nat. Commun..

[42] Tan S.K., Cebrik D., Plotnik D., Agostini M.L., Boundy K., Hebner C.M., Yeh W.W., Pang P.S., Moya J., Fogarty C. (2024). A Randomized, Placebo-Controlled Trial to Evaluate the Safety and Efficacy of VIR-2482 in Healthy Adults for Prevention of Influenza A Illness (PENINSULA). Clin. Infect. Dis..

[43] Beukenhorst A.L., Rice K.L., Frallicciardi J., Koldijk M.H., Boudreau C.M., Crawford J., Cornelissen L.A.H.M., da Costa K.A.S., de Jong B.A., Fischinger S. (2025). Intranasal administration of a panreactive influenza antibody reveals Fc-independent mode of protection. Sci. Rep..

[44] Beukenhorst A.L., Frallicciardi J., Rice K.L., Koldijk M.H., de Mello J.C.M., Klap J.M., Hadjichrysanthou C., Koch C.M., da Costa K.A.S., Temperton N. (2024). A pan-influenza monoclonal antibody neutralizes H5 strains and prophylactically protects through intranasal administration. Sci. Rep..

[45] Döhrmann S., Levin J., Cole J.N., Borchardt A., Amundson K., Almaguer A., Abelovski E., Grewal R., Zuill D., Dedeic N. (2025). Drug–Fc conjugate CD388 targets influenza virus neuraminidase and is broadly protective in mice. Nat. Microbiol..

[46] Döhrmann S., Levin J., Cole J.N., Borchardt A., Amundson K., Almaguer A., Abelovski E., Grewal R., Zuill D., Dedeic N. (2024). CD388: A universally protective Drug-Fc Conjugate that targets influenza virus neuraminidase. bioRxiv.

[47] Rojas R.E., Equils O., Villacian J., Mann A., van Duijnhoven W., Vingerhoets J., Baguet T., Wang S.-S., Anandakumar A., Anger A. (2025). Prophylactic Efficacy of CD388, a Novel Drug–Fc Conjugate, in a Human Influenza A/H3N2 Virus Challenge Model: A Randomized, Controlled Phase 2a Study. Clin. Infect. Dis..

[48] Fiore A.E., Fry A., Shay D., Gubareva L., Bresee J.S., Uyeki T.M. (2011). Antiviral agents for the treatment and chemoprophylaxis of influenza: Recommendations of the Advisory Committee on Immunization Practices (ACIP). Morb. Mortal. Wkly. Rep..

[49] O’lEary S.T., Campbell J.D., Ardura M.I., Bryant K.A., Caserta M.T., Espinosa C., Frenck R.W., Healy C.M., John C.C., Committee on Infectious Diseases (2024). Recommendations for Prevention and Control of Influenza in Children, 2024–2025: Policy Statement. Pediatrics.

[50] O’lEary S.T., Campbell J.D., Ardura M.I., Bryant K.A., Caserta M.T., Espinosa C., Frenck R.W., Healy C.M., John C.C., Committee on Infectious Diseases (2024). Recommendations for Prevention and Control of Influenza in Children, 2024–2025: Technical Report. Pediatrics.

[51] Uyeki T.M., Bernstein H.H., Bradley J.S., Englund J.A., File T.M., Fry A.M., Gravenstein S., Hayden F.G., Harper S.A., Hirshon J.M. (2019). Clinical Practice Guidelines by the Infectious Diseases Society of America: 2018 Update on Diagnosis, Treatment, Chemoprophylaxis, and Institutional Outbreak Management of Seasonal Influenzaa. Clin. Infect. Dis..

[52] Dardas L.A., Al-Leimon O., Jaber A.R., Saadeh M., Al-Leimon A., Al-Hurani A., Jaber A.-R., Aziziye O., Al-Salieby F., Aljahalin M. (2023). Flu Shots Unveiled: A Global Systematic Review of Healthcare Providers’ Uptake of, Perceptions, and Attitudes toward Influenza Vaccination. Vaccines.

[53] Uyeki T.M., Bernstein H.H., Bradley J.S., Englund J.A., File T.M., Fry A.M., Gravenstein S., Hayden F.G., Harper S.A., Hirshon J.M. (2019). Clinical Practice Guidelines by the Infectious Diseases Society of America: 2018 Update on Diagnosis, Treatment, Chemoprophylaxis, and Institutional Outbreak Management of Seasonal Influenzaa. Clin. Infect. Dis..

[54] Butler C.C., van der Velden A.W., Bongard E., Saville B.R., Holmes J., Coenen S., Cook J., Francis N.A., Lewis R.J., Godycki-Cwirko M. (2020). Oseltamivir plus usual care versus usual care for influenza-like illness in primary care: An open-label, pragmatic, randomised controlled trial. Lancet.

