# Supplementary material for: Advancing Influenza Prevention: The Case for Pre-Exposure Prophylaxis (PrEP)
Source: Vaccines (Basel). 2026 Jul 30;14(8):666. doi: 10.3390/vaccines14080666 (PMC13517930; doi:10.3390/vaccines14080666)
Supplement: Supplementary file 1 [file vaccines-14-00666-s001.zip › vaccines-4405309-supplementary.pdf]

Title: Advancing Influenza Prevention: The Case for Pre-Exposure Prophylaxis (PrEP)

Supplementary Material

Agent Based Model

Synthetic Population

The agent-based model (ABM) relies on a synthetic population of the greater New York City area as of 2017. This synthetic population is constructed using population information from the American Community Survey (ACS) Public Use Microdata Sample (PUMS) 1-year (2016-17) and 5-year (2013-17) datasets. It also uses school enrollment information from the National Center for Educational Statistics (NCES) Common Core of Data (CCD) and Private School Survey (PSS) data from 2017-18 and commuter/workplace information from and the Census County Business Patterns (CBP) and Census Transportation Planning Product (CTPP) 2012-16 dataset.

Information about household composition, including age, sex, education and employment status of household members, and household size, is pulled from the 2013-17 ACS survey. Rejection sampling over households by weight is then used to generate a dataset of households that matched the distribution of ACS PUMS 2016-17 data. The resulting dataset includes census tract, pseudo-household ID, and demographic information for each individual in the synthetic population. Public school students are assigned to schools on a county-by-county basis, with school enrollment occurring in a random order so that students are assigned to the nearest school with available enrollment capacity. Private school students are assigned to schools in either their home or neighboring county. Any remaining students not assigned to a school are assigned to an eligible nearby school using random sample assignment. Workplaces for a given census tract are generated using the distribution of workplace sizes pulled from CBP data. Workers are assigned a census tract for work using CTPP data to capture the number of workers who work in their home census tract or neighboring census tracts. Finally, workers are then assigned to workplaces in their work census tract proportional to the size of the workplaces [1].

These data are combined to create a dataset that identifies individuals in the population by household, community, and optional school and work information. Individuals are then re-categorized into one of three age groups: minors, adults and seniors, with associated age ranges 0-17, 18-64 and 65+. This synthetic population is then used as an input into the ABM, as described below[2 3].

GCM-EpiNext

GCM is a discrete event simulation engine for building pathogen transmission models (https://github.com/hhs/aspr-8). EpiNext is an ABM for directly transmitted pathogens and built on top of the GCM engine. For the current work, we consider a susceptible, exposed, infectious and recovered compartmental model (SEIR), in which the duration of the latent and infectious periods (residence times in the E and I compartments, respectively) are drawn randomly from a distribution for each individual. These distributions are not assumed to vary by age[4]. Individuals in the infected compartment may or may not develop symptoms according to pre-specified probabilities that are also impacted by the effects of MCMs. See Supplementary Table 1 for parameter values.

Supplementary Table S1

Parameter	Assumptions	Reference
Latent period	Exponentially distributed with a mean of 2.5 days	Cori et al, Saito et al.; Boëlle et al.
Infectious period	Exponentially distributed with a mean of 1.5 days	Cori et al, Saito et al; Boëlle et al.
Fraction Symptomatic	0.7	Bautista et al.; Leung et al.; Tsang et al.
Basic reproductive number	Three pandemic scenarios considered with basic reproductive numbers: 1.8 (1918), 1.64 (1968) and 1.4 (2009).	Biggerstaff el al.
Infectious contact rate by age group	Calibrated to study.	Prem et al, 2021
Infectious contact setting selection probability	Calibrated to study.	Prem et al., 2021
Infectious contact target age group	Proportionate mixing.	

Parameter	Assumptions	Reference
selection probability		

In the model, infectious individuals in the synthetic population make contact with other individuals stochastically, with the mean contact rates shown in Supplementary Table 2. The relative contact rates by age were chosen to match the overall number of contacts per day from contact studies (Prem et al, 2021). An overall normalizing multiplier to all age groups is applied so as to match the basic reproductive numbers for the three influenza pandemics of 1918, 1968 and 2009, in decreasing order of transmissibility (Biggerstaff et al, 2014). These normalizing multipliers were determined by repeatedly simulating stochastic realizations over a range of multiplier values and selecting the three values that resulted in the basic reproductive numbers listed in Supplementary Table 1, after averaging [5 6 7].

Contact with other individuals may occur in different settings, depending upon which groups the individual belongs to. We consider four overall settings: home, school, work and community (individuals within the same census tract). All individuals are associated with a home and a community, but they may or may not be associated with schools and workplaces. The probability of choosing a given contact setting is informed by the literature (Prem et al, 2021). Once a setting is chosen, contact is made with each individual in the group with equal probability, regardless of their age group. In addition, community contacts have a certain probability of occurring outside of an individual's census tract, up to a certain distance, to reflect long distance travel/interactions.

Supplementary Table S2

Pandemic Scenario	Age Group	Mean Infectious Contact Rate (days^{-1})
2009	< 18	1.34
2009	18 – 64	1.10
2009	65+	0.67
1968	< 18	1.70
1968	18 – 64	1.40
1968	65+	0.85

1918	< 18	1.83
1918	18 – 64	1.50
1918	65+	0.91

Parameter Assumptions

We assume epidemiological parameter values as listed in Supplementary Table 1.

Vaccine parameters are listed in Supplementary Table 3. In our ABM, we assume a 2-dose vaccine where doses are administered 3 weeks apart. Vaccine recipients are chosen randomly across the population. Protection conferred by the vaccine ramps up linearly, starting immediately after the second dose and reaching full efficacy after 2 weeks (thus, 4 weeks after the first dose). We assume negligible waning over the course of a single season/epidemic wave. From the values in Supplementary Table 3, we obtain a vaccine efficacy against symptomatic influenza of 0.58.

Efficacy values for the PrEP product are measured with respect to those of an available vaccine (Supplementary Table 3). The combined efficacy against symptomatic influenza for individuals receiving PrEP is 70% relative to vaccinated individuals, and a single dose of PrEP is assumed to be protective for a full season/epidemic wave with negligible waning. We model PrEP as having no ramp up period and thus provides full protection immediately after the single dose. PrEP recipients are chosen randomly across the population and the complete PrEP stockpile is disbursed early, when total infections are still negligible.

Supplementary Table S3

MCM parameter	Assumptions	Reference
Vaccine course	Two injections separated by 2 weeks	
Vaccine efficacy against infection (after linear ramp up starting after the 2nd dose)	40%	Basta et al.; Belongia et al.
Vaccine efficacy against clinical symptoms, given infection (after linear ramp up starting after the 2nd dose)	30%	Basta et al.; Belongia et al.

Combining PrEP and Vaccination for Mitigation

We ran additional simulations to model the combined effect of the TPP-optimal PrEP product and a vaccine. We assume vaccination is available quickly, with doses available 45 days after the first one hundred infections are present in the population, and that there exists sufficient inventory to vaccinate either 25% or 50% of the population. Moreover, we assume that there is capacity to apply vaccine doses equivalent to 2% of the population per week. In the case of the synthetic population of 19.5M that we use, this results in about 60k vaccinations per day, with vaccination inventories disbursed by approximately days 200 and 350 after the first 100 infections for the 25% and 50% inventory scenarios, respectively. There is low stochastic variability at the parameter values used in this model; throughout this section we show results for a single replication for each set of parameter values.

We calculate the fraction of infections averted, relative to the unmitigated baseline, and whether these are attributable to either PrEP or vaccine. We consider vaccine coverages of 0%, 25% or 50% and PrEP coverages from 0% to 50% in steps of 10%. Figure S1 shows that despite the quick deployment of vaccines in the population, vaccines on their own would provide negligible mitigation at any of the transmissibility levels considered. For vaccines to have a significant impact under the current production and distribution assumptions, other means of mitigation are needed to delay the epidemic wave. When vaccines are used in conjunction with a sizable and fast deployment of PrEP, the use of PrEP countermeasures is potentially able to delay the epidemic peak sufficiently to extend the time window during which vaccines can have an effect (Fig. 1, main text) [8,9 10]. The time lag in the epidemic peak is larger as the proportion of the population receiving PrEP grows; a consequence of this is that infections averted by vaccination increase as the PrEP coverage goes up (Fig. S1). However, vaccines are not distributed quickly enough for the vaccine inventory equivalent to 50% of the population to make a difference relative to the one covering only 25% (Fig. S1, center and right columns).

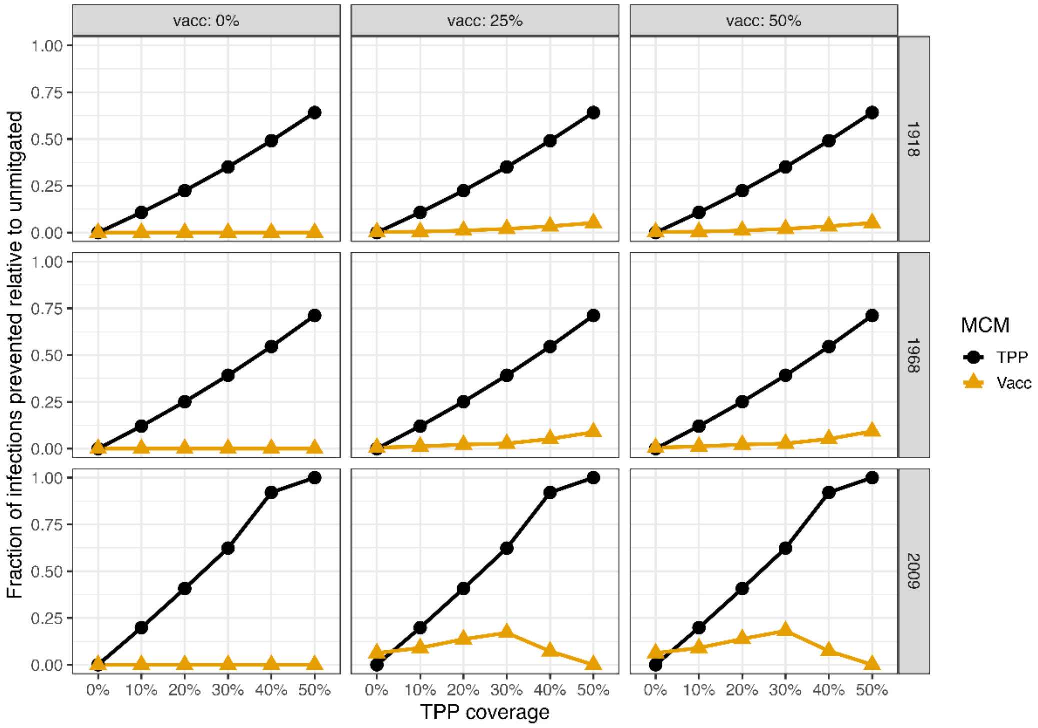

Figure S1: fraction of infections averted, relative to an unmitigated baseline, as a function of TPP coverage (x-axis), vaccine coverage (panels along columns), virus transmissibility scenario (panels along rows) and by how they are attributed by MCM (colors and marker shape).

References

1. Prem K, Zandvoort KV, Klepac P, et al. Projecting contact matrices in 177 geographical regions: An update and comparison with empirical data for the COVID-19 era. PLoS Comput Biol 2021;17(7):e1009098. (In eng). DOI: 10.1371/journal.pcbi.1009098.
2. Saito MM, Hirotsu N, Hamada H, et al. Reconstructing the household transmission of influenza in the suburbs of Tokyo based on clinical cases. Theor Biol Med Model 2021;18(1):7. (In eng). DOI: 10.1186/s12976-021-00138-x.
3. Tsang TK, Wang C, Fang VJ, et al. Reconstructing household transmission dynamics to estimate the infectiousness of asymptomatic influenza virus infections. Proceedings of the National Academy of Sciences 2023;120(33):e2304750120. DOI: doi:10.1073/pnas.2304750120.
4. Leung NH, Xu C, Ip DK, Cowling BJ. Review Article: The Fraction of Influenza Virus Infections That Are Asymptomatic: A Systematic Review and Meta-analysis. Epidemiology 2015;26(6):862–72. (In eng). DOI: 10.1097/ede.0000000000000340.

5. Cori A, Valleron AJ, Carrat F, Scalia Tomba G, Thomas G, Boëlle PY. Estimating influenza latency and infectious period durations using viral excretion data. Epidemics 2012;4(3):132–138. DOI: https://doi.org/10.1016/j.epidem.2012.06.001.
6. Boëlle P-Y, Ansart S, Cori A, Valleron A-J. Transmission parameters of the A/H1N1 (2009) influenza virus pandemic: a review. Influenza and Other Respiratory Viruses 2011;5(5):306–316. DOI: https://doi.org/10.1111/j.1750-2659.2011.00234.x.
7. Biggerstaff M, Cauchemez S, Reed C, Gambhir M, Finelli L. Estimates of the reproduction number for seasonal, pandemic, and zoonotic influenza: a systematic review of the literature. BMC Infect Dis 2014;14:480. (In eng). DOI: 10.1186/1471-2334-14-480.
8. Bautista E, Chotpitayasunondh T, Gao Z, et al. Clinical aspects of pandemic 2009 influenza A (H1N1) virus infection. N Engl J Med 2010;362(18):1708–19. (In eng). DOI: 10.1056/NEJMra1000449.
9. Belongia EA, Simpson MD, King JP, et al. Variable influenza vaccine effectiveness by subtype: a systematic review and meta-analysis of test-negative design studies. Lancet Infect Dis 2016;16(8):942–51. (In eng). DOI: 10.1016/s1473-3099(16)00129-8.
10. Basta NE, Halloran ME, Matrajt L, Longini IM, Jr. Estimating influenza vaccine efficacy from challenge and community-based study data. Am J Epidemiol 2008;168(12):1343–52. (In eng). DOI: 10.1093/aje/kwn259.
